# Robotic minimally invasive inguinal hernia repair with the Dexter robotic system™: A prospective multicenter clinical investigation

**DOI:** 10.1007/s00464-024-11361-1

**Published:** 2024-11-14

**Authors:** Lukas Gantner, Hubert Mignot, Julius Pochhammer, Felix Grieder, Stefan Breitenstein

**Affiliations:** 1 Department of Visceral and Thoracic Surgery, Kantonsspital Winterthur, Brauerstrasse 15, 8400 Winterthur, Switzerland; 2 Department of General Surgery, Centre Hospitalier de Saintes, Saintes, France; 3 Clinic for General, Visceral, Thoracic, Transplant and Pediatric Surgery, Universitätsklinikum Schleswig-Holstein, Kiel, Germany

**Keywords:** Hernia repair, Herniorrhaphy, Inguinal hernia repair, Robot assisted surgery, New robotic system, Dexter robotic system

## Abstract

**Background:**

Robot-assisted transabdominal preperitoneal inguinal hernia repair (rTAPP) has been established with various robotic platforms. The Dexter robotic system is an open platform consisting of a sterile surgeon’s console, two robotic instrument arms, and one robotic endoscope arm. This study aimed to confirm the perioperative and early postoperative safety and clinical performance of the Dexter system in patients undergoing primary transperitoneal inguinal hernia repair.

**Methods:**

The primary objectives of this multicenter study conducted at three centers in France, Germany, and Switzerland were to document the successful completion of rTAPP procedures and the occurrence of serious adverse events (Clavien–Dindo grades III–V), device-related events up to 30 days post-surgery. The procedures were performed by three surgeons with varying levels of experience in robotic systems.

**Results:**

50 patients with a median age of 62.5 years (IQR 51.0–72.0) and BMI of 25.1 kg/cm^2^ (IQR 23.5–28.7), respectively, underwent inguinal hernia repair (33 unilateral, 17 bilateral). All surgeries were successfully completed using three standard laparoscopy trocars. There were no conversions to open surgery, intraoperative complications or device deficiencies. The median skin-to-skin operative time was 50 min (IQR 45–60) for unilateral hernias and 96 min (IQR 84–105) for bilateral hernias. The median console time was 30 min (IQR 26–41) for unilateral and 66 min (IQR 60–77) for bilateral hernias. Twenty-six patients were discharged on the day of surgery, and 22 on postoperative day 1.

**Conclusion:**

This study confirmed the use of the Dexter system in rTAPP was feasible and safe in multicenter cohorts, with operative times consistent with the literature on other robotic platforms. Our data demonstrated the accessibility of this new robotic approach, even when adopted by surgeons new to robotics. The Dexter system emerged as a valuable device in the hernia repair toolkit for both experienced robotic surgeons and those new to the field.

**Supplementary Information:**

The online version contains supplementary material available at 10.1007/s00464-024-11361-1.

Globally, inguinal hernia repairs are among the most common surgical procedures, with more than 20 million performed each year. The lifetime risk for men ranges between 27 and 43%, while for women it is between 3 and 6% [1–3]. Inguinal hernias typically require surgery for definitive treatment. Although a minority of patients might not show symptoms initially, a watchful waiting approach still results in surgery for approximately 70% of these patients within 5 years [2, 4].

An optimal inguinal hernia repair technique should prioritize safety and minimize the risk of recurrence, ensure minimal acute postoperative pain and facilitate a quick return to daily activities. Additionally, it should have a short learning curve to enhance safety and quality during the initial learning phase before reaching surgical proficiency. The Lichtenstein technique remains the expert consensus for open hernia repair, due to its safety, with low recurrence rates and affordability, making it accessible globally [2, 5–7]. Minimally invasive laparoscopic repair is equal to open approaches in recurrence rate and has a similar safety profile, and it has additionally demonstrated patient benefits in the form of reduced wound complications [8, 9], diminished acute and chronic inguinal pain and faster recovery [2, 8, 10–13]. Minimally invasive techniques should therefore be used if the surgical expertise is available [2].

The drawbacks of laparoscopic hernia repair include a steep learning curve, a higher likelihood of recurrence and complications at the outset of a surgeon’s experience and increased costs. The learning curve for endoscopic techniques is estimated to be 50–100 cases, with the first 30–50 cases being the most critical learning phase, whereas the Lichtenstein technique is easier to teach and replicate at all levels [2, 7, 14, 15]. Studies have indicated that surgeons who have conducted fewer than 50 laparoscopic hernia procedures in the preceding year experienced higher hernia recurrence rates compared to the open technique [7, 15].

Robotic technology has revolutionized the field of surgery, offering precision, enhanced dexterity with articulating instrumentation, three-dimensional visualization, and improved ergonomics for the surgeon. While urologists were the first to employ robotic systems for inguinal hernia repairs as a supplementary procedure to prostatectomies [16], general surgeons have rapidly incorporated this innovative technique into their toolkit. The earliest series of robotic-assisted hernia repairs as a standalone procedure emerged in 2015 [17]. Since that time, the adoption of robotic-assisted hernia repair has increased significantly, accompanied by an increase in studies demonstrating its feasibility [18] and comparable results to standard laparoscopy in terms of postoperative results and safety profile [18–27]. Some have criticized longer operation times and the unnecessary higher cost [22, 23].

New robotic systems have recently entered the market demonstrating their feasibility and safety [28]. The Dexter robotic system (Distalmotion, Epalinges, Switzerland) received CE marking in 2020 for use in gynecological, urological, and general surgical procedures. The Dexter system can be integrated into the established laparoscopic workflow.

The feasibility and safety of the Dexter system have previously been documented in prostatectomy [29, 30], gynecology [31, 32], and general visceral surgery [33, 34]. We conducted the first prospective multicentric investigation to confirm the perioperative and early postoperative safety and clinical performance of the Dexter system in patients undergoing primary transperitoneal inguinal hernia repair.

## Materials and methods

### Study design

The prospective, single-arm, multicenter study was performed at three centers in France, Germany, and Switzerland. The study was conducted according to the Declaration of Helsinki, ISO 14155:2020 and 21 CFR 812.28. The study protocol approval by the relevant Ethical Committees was collected according to local requirements and registered in the ClinicalTrials.gov database (NCT05873582) before the start of the recruitment. Informed consent was obtained from all participants. The primary objectives of the study were to document the successful completion of the robotic transabdominal preperitoneal inguinal hernia repair (rTAPP) procedure and to collect data on the occurrence of serious adverse events (Clavien-Dindo grades III-V), device-related events, and other adverse events perioperatively and up to 30 days post-surgery. The procedures were performed by three surgeons with varying levels of experience in laparoscopy (LAP) and robotic-assisted surgical (RAS) procedures. Surgeon 1 had 19 years of LAP experience and 5 years of RAS experience with over 290 procedures completed prior to the Dexter training. Surgeon 2 had 35 years of LAP and no prior RAS experience. Surgeon 3 had 7 years of LAP, and limited prior RAS experience. All surgeons received the Dexter training and had performed approximately 40 surgeries with the Dexter system prior to study enrollment.

### Patient population

Inclusion criteria were subjects aged 18 years or older scheduled to undergo elective robot-assisted laparoscopic surgery for primary unilateral or bilateral inguinal hernia repair, and willing to participate in the 30-day follow-up. The exclusion criteria were BMI > 40, contraindications for endoscopic procedures, need for robotic stapling, advanced energy delivery, ultrasound, cryoablation, and microwave energy delivery, bleeding diathesis, pregnancy, pacemakers or internal defibrillators, and objection to the collection of their data for research purpose.

Procedure-specific exclusion criteria included subjects with emergent inguinal hernia repair, a history of major abdominal or pelvic surgery, strangulated and incarcerated inguinal hernia, large scrotal hernia, prior prostatectomy, hysterectomy, or any other uterine procedures, prior radiotherapy for prostatic cancer and previous preperitoneal mesh placement on the site of the planned inguinal hernia repair.

### Robotic system

The Dexter robotic system (Distalmotion SA, Epalinges, Switzerland) comprises of a sterile surgeon console, two patient carts with robotic arms, one endoscope arm that can accommodate any endoscopic system and five wristed instruments with seven degrees of freedom. Its compact profile allows the surgeon and the surgical staff to move freely around the patient. The robotic arms can be folded back in a LAP mode function, allowing the sterile surgeon to switch between laparoscopic and robotic mode in a matter of seconds without re-docking the robot. The single-use instruments employed for the rTAPP were the monopolar scissors, the Johann grasper, and the needle holder.

### Surgical technique

**Fig. 1:**
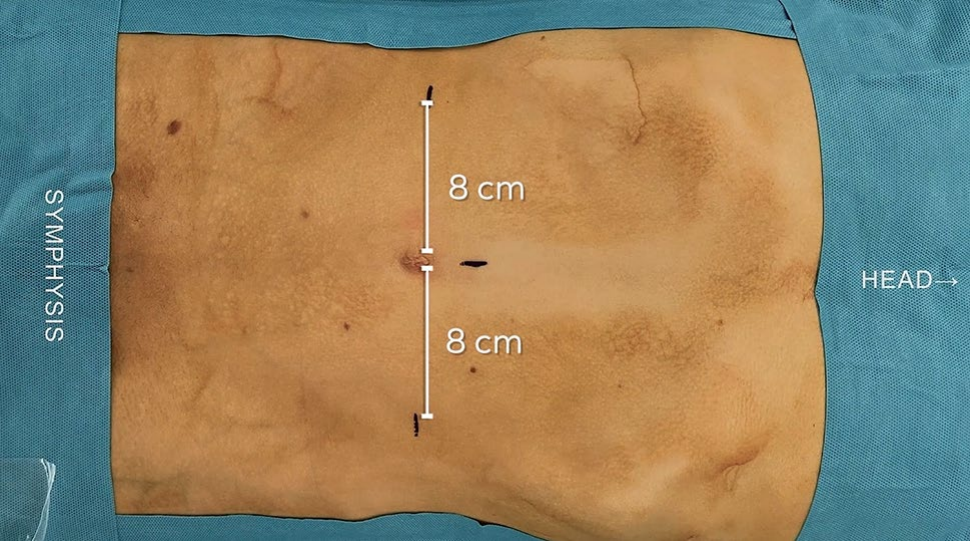
Trocars placement for inguinal hernia repair with the rTAPP method with the Dexter robotic system

**Fig. 2:**
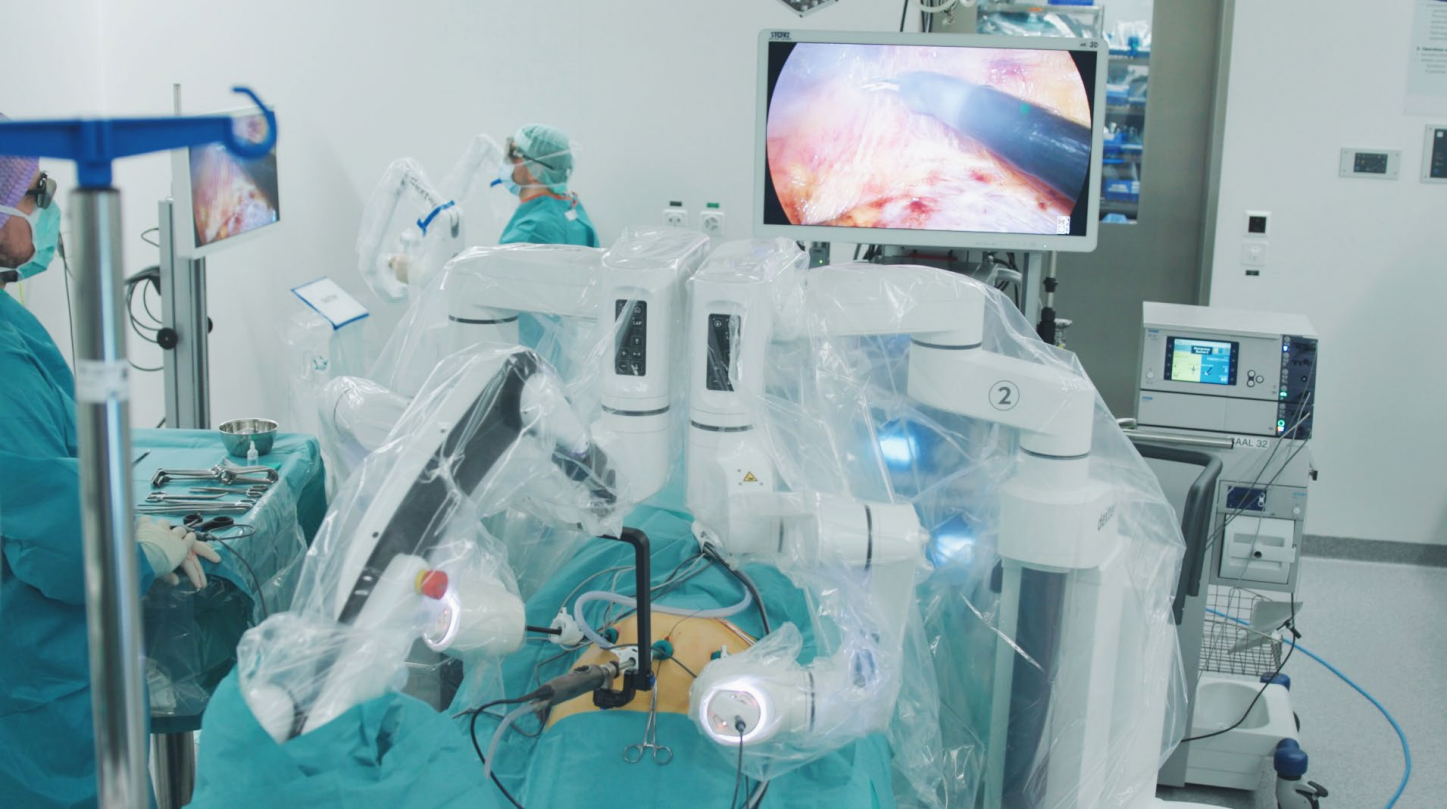
Photo of the operating room set up. The surgeon remains sterile at the draped console (upper left), while controlling the two robotic arms. Only one scrubbed nurse is assisting during the surgery (front)

The patients were placed in a supine position with a 7 to 20° Trendelenburg tilt. One supraumbilical port was placed for the 3D endoscope camera and two 10/11/12 mm robotic ports were placed at a distance of 8–9 cm from the umbilicus at the umbilical level (Fig. 1).The patient carts were placed on either side of the patient bed, with the endoscope arm positioned at the cephalic level (Fig. 2). The robotic arms were docked to the trocars by aligning the instrument’s remote center of motion with the trocar under direct visualization. The monopolar scissors were inserted in the right arm and the Johann grasper in the left arm. The peritoneal flap was created, and the anatomical landmarks were identified followed by reduction of the hernia sac and its content. The mesh was inserted laparoscopically through the robotic instrument trocar and robotically placed over the hernia defect and secured. No fixation was used in cases where self-gripping mesh or fixation-free mesh was used. Finally, the scissors in the right arm were replaced by a needle holder and the peritoneal flap was closed with sutures, the robotic instruments were removed, and the incisions were closed. The principal steps of rTAPP with Dexter are shown in the video (Supplementary Video S1).

### Data collection and statistical analysis

Safety and performance data were collected intra-operatively, and patients were followed up for 30 days after the surgery. The operative time was defined as time from the first skin incision to the last suture. The docking time was measured in minutes, starting from the moment the order was given to move the patient carts near the bed and ending when the last incision pointer was removed or the endoscope was attached to the camera arm, whichever occurred later. The console time was defined as the total time that the robot was in use in minutes.

Adverse events were reviewed and adjudicated by an independent Clinical Event Committee. Descriptive statistics were used in this study and the performance of the Dexter system was assessed quantitatively by its conversion rate. For descriptive statistics, Median and Interquartile range (IQR) were used to present continuous data. Numbers and percentages were used for categorical variables. The Kruskal–Wallis test was used to compare the operative times among surgeons. The significance level (p-value) was determined at 0.05. Data were analyzed using StataCorp. (2023. Stata Statistical Software: Release 18. College Station, TX: StataCorp LLC).

## Results

From June to October 2023, 50 patients were operated for primary inguinal hernia (33 unilateral, 17 bilateral). The majority of the patients were male (94%) with a median age of 62.5 years (IQR 51.0–72.0) and BMI 25.1 kg/cm^2^ (IQR 23.5–28.7) (Table 1).

**Table 1:** Patient characteristics

Characteristics	*N* = 50
Age (years), median (IQR)	62.5 (51.0–72.0)
Gender	
Male	94.0% (47/50)
BMI (kg/m^2^), median (IQR)	25.1 (23.5–28.7)
ASA status % (n/N)	
ASA I	26.0% (13/50)
ASA II	66.0% (33/50)
ASA III	8.0% (4/50)
Hernia type, n (%)	
Unilateral	33 (66%)
Bilateral	17 (34%)
Left side	29 (43.3%)
Right side	38 (56.7%)
Hernia location, n (%), *N* = 67	
Lateral/indirect	31 (46.3)
Medial/direct	6 (9.0)
Femoral	0 (0)
Mixed L/F	10 (14.9)
Mixed L/M	17 (25.4)
Mixed M/F	1 (1.5)
Mixed M/L/F	2 (3.0)

*IQR* interquartile range

Each procedure was performed robotically as planned with no conversions to open surgery. No intraoperative complications or device deficiencies were observed. No additional trocar sites were required during the procedures. One conversion to conventional laparoscopy was necessary due to port placement too far medial, impeding access to the surgical area (Table 2). No additional port placement was required in this patient to pursue laparoscopic surgery. The median skin-to-skin operative time was 50.0 min (IQR 45.0–60.0) for unilateral hernia repair and 96.0 min (IQR 84.0–105.0) for bilateral hernia repair. The median robotic console time was 30.0 min (IQR 26.0–41.0) for unilateral hernia and 66.0 min (IQR 60.0–77.0) for bilateral hernia repairs. Median docking time was 4.0 min (IQR 3.0–5.0). In 37.3% of the cases, the mesh was fixated with a suture and in 4.5% with a tack, while 31.3% of the meshes used were self-gripping and did not need to be fixated. In 26.9% of the cases, anatomical shaped, fixation-free 3D mesh was used (Table 2). Twenty six (52%) patients were discharged on the same day of the surgery, and 22 (44%) patients discharged on postoperative day 1 (POD 1) according to local clinical practice. There were 12 postoperative adverse events during the 30-day follow-up period, with only one classified as serious (Clavien-Dindo grade II) (Table 3). One subject was re-hospitalized due to a small intestine occlusion with possible adhesion in the right iliac fossa. The event resolved without any sequelae on postoperative day 7. None of the adverse events were Clavien-Dindo grades III–V. A breakdown of the surgical procedure time per surgeon was performed to evaluate the impact of previous surgical experience on the use of the Dexter system (Table 4). Comparison of total procedure time and console time among the three surgeons showed a significant difference for unilateral but not for bilateral hernia repair.

**Table 2:** Operative variables for the inguinal hernia repairs

Parameter	Value
Conversion to open, n (%)	0 (0)
Conversion to LAP, n (%)	1 (2)
Operative time (skin-to-skin) (min), median (IQR)	
Unilateral	50.0 (45.0–60.0)
Bilateral	96.0 (84.0–105.0)
Console time (min), median (IQR)	
Unilateral	30.0 (26.0–41.0)
Bilateral	66.0 (60.0–77.0)
Docking time (min), median (IQR)	4.0 (3.0–5.0)
Length of Hospital stay (days), median (IQR)	0.0 (0.0 – 1.0)
Same day discharge, n (%)	26 (52%)
POD1 discharge, n (%)	22 (44%)
POD2 discharge, n (%)	2 (4%)
Return to activities (days), median (IQR)	5.0 (1.0–14.0)
Estimated blood loss (mL), median (IQR)	0.0 (0.0–10.0)
Intraoperative complications, n (%)	0 (0)
Mesh fixation method, n (%)	
Suture	25 (37.3)
Self-gripping	21 (31.3)
Fixation-free, 3D-shaped	18 (26.9)
Tacks	3 (4.5)

*LAP* laparoscopic surgical procedure, *POD* postoperative day, *IQR* interquartile range

**Table 3:** Postoperative outcomes at 30 days

Outcomes	*N* = 50
Adverse Events, n (%)	12 (24)
Anesthesia complications	1 (2)
Hematoma	3 (6)
Persistent groin pain	1 (2)
Seroma	3 (6)
Trocar specific complication	1 (2)
Other	3 (6)
Clavien-Dindo classification	
Grade I	11 (22)
Grade II	1 (2)
Grade III – V	0 (0)
Mortality, n (%)	0 (0)
Rehospitalization, n (%)	1 (2)
Reoperation, n (%)	0 (0)

**Table 4:** Operative time and console time break down per surgeon

	Surgeon 1	Surgeon 2	Surgeon 3	p value^a^
Procedure time (min)				
Unilateral				
N	7	16	10	
Median (IQR)	46.0 (38.0–54.0)	49.0 (43.5–56.0)	68.0 (56.0–76.0)	0.013
Bilateral				
N	3	4	10	
Median (IQR)	101.0 (78.0–109.0)	90.0 (65.5–100.0)	97.0 (87.0–108.0)	0.594
Console time (min)				
Unilateral				
N	7	16	10	
Median (IQR)	30.0 (22.0–34.0)	28.5 (23.0–37.5)	49.5 (38.0–56.0)	0.008
Bilateral				
N	3	4	10	
Median (IQR)	62 (54.0–83.0)	61.5 (41.0–66.5)	73.5 (41.0–108.0)	0.299

^a^Calculated with the Kruskal–Wallis test*; IQR* Interquartile Range

## Discussion

Since their introduction, surgical robots have increased the popularity of rTAPP by providing surgeons with enhanced dexterity, precision, and visualization [35–38]. These advantages translate into patient outcome benefits such as shorter time spent in the recovery room, shorter hospital stay, and reduced postoperative pain [39–41]. rTAPP has, however, been associated with longer operative times and higher costs compared to standard laparoscopic techniques [42, 43], in particular demonstrated in the head-to-head comparison between the techniques in the RIVAL trial [22, 23]. The Dexter robotic system is an innovative and simple modular device that integrates into the already existing workflow of any laparoscopic OR. We have hereby reported the first prospective inguinal hernia repair study conducted with the Dexter system. The primary endpoints in this study were the conversion rate to open or laparoscopic surgery and the occurrence of serious adverse events defined as Clavien-Dindo III-V. The low minor complication rate, the absence of serious adverse events, and the positive clinical performance confirm the safety and effectiveness of the Dexter robotic system for rTAPP. In the 50 procedures performed, there were no conversions to open surgery. In the literature, the conversion to open surgery with the Da Vinci system ranged from 0 to 1.4% [17, 40, 41, 44, 45], although the conversion rate in patients with obesity was higher (3.2%). With the Senhance system, the reported conversion rates were 1.5% to open surgery and 3% to laparoscopic surgery [46]. The reported conversion rates of more recent systems were less reliable, with anecdotal reports ranging from 0 to 10% [47–49]. In our study, one procedure was converted to laparoscopic due to a suboptimal placement of the trocars that hindered access to the surgical area. The port placement used with the Dexter system is the same as the one used in laparoscopic surgery, which facilitates the switch between laparoscopic and robotic mode during the surgery. The transition to laparoscopic mode was smooth and required no additional trocar placement to finalize the procedure. The surgeon, scrubbed in at the sterile surgeon console during the robotic procedure, could intervene at the operating table within seconds. The operative time of the converted bilateral procedure was 101 min, which is in the same range as for the whole cohort, further demonstrating the flexibility of the Dexter system.

There were no intraoperative complications and no serious complications (Clavien-Dindo III-V) within the study period. These results are in line with complication rates described with similar systems. Reported intraoperative complications [40, 41, 45, 46, 50–52] and serious early postoperative complications are generally rare in rTAPP [41, 45, 46].

The majority of patients were discharged on the day of the procedure, with a short hospital stay for the others. In Europe, reimbursement practices for inguinal hernia procedures sometimes results in overnight stays, especially for bilateral hernia repair, to ensure sufficient coverage. This is why many study participants were discharged after one day.

While some of the literature on robotic repair indicates longer operative times compared to laparoscopic surgery [39], some studies have presented comparable operative times for the two techniques [19]. In general, the reported operative times range from 54 to 103 min for unilateral inguinal hernia repair [17, 25, 40, 43, 51, 53–57] and from 59 to 147 min for bilateral inguinal hernia repair [25, 40, 43, 51, 55–57]. Studies that compare operative times early in the learning curve to later in the learning curve show improved surgeon performance later in the learning curve [51, 58]. The variability of the learning phase duration may impact the performance reported in comparative studies studying operative times. The OR times in our study aligned with those reported for other surgical robots. All procedures were performed by three surgeons with varying experience in laparoscopy and robotic surgery prior to Dexter training. Surgical time comparisons showed some variability for the unilateral inguinal hernia repair, but performance was similar for bilateral cases. This suggests that all surgeons reached comparable performance, regardless of prior experience. These results suggest that the learning curve for the Dexter system is short and that surgical experience is rapidly translated into Dexter proficiency. A surgeon who is trained in laparoscopic surgery will benefit from the familiarity of the port placement when transitioning into robotic surgery with the Dexter system. In our study, the variability of operative times may also be due to differences in mesh types and fixation, as the faster surgeon used a self-fixating mesh.

The port placement and the large working space around the patient bed, particularly when the robot is in laparoscopic mode, with the robotic arms folded, allow certain steps of the procedure to be performed laparoscopically when it is more time-efficient or according to the surgeon’s preference. As the surgeon remains sterile throughout the procedure, the transfer between the patient bedside and the surgeon console is instantaneous and does not necessitate additional scrubbing. For example, the insertion of the mesh may be performed by the sterile surgeon without the need for an assisting surgeon at the bedside [34].

We conducted a prospective, multi-institutional study on the use of the Dexter system for inguinal hernia repair. The study however has several limitations. Only elective, primary repairs were included, limiting the generalizability of the results. The single-arm design lacked a control group, and comparisons were made to literature data with similar robotic systems, which is generally based on retrospectively collected data. The small sample size of 50 patients and the short follow-up period limited analysis to early postoperative outcomes. Larger, randomized studies with longer follow-up are needed for conclusive results. The reimbursement constraints in Europe required some overnight stays, increasing the average length of stay. Variations in mesh types, fixation methods, and surgeon’s previous rTAPP experience with the Dexter system also impacted the results.

Further directions include investigations of the learning curve of the Dexter system, skills transferability, workflow and operating room communication and efficiency as advantages of the open platform as well as ergonomic studies. Analysis of data from real world use of the Dexter system would provide additional conclusions on the safety and clinical performance of the Dexter system.

## Conclusion

The use of the Dexter system in rTAPP was feasible and safe in multicenter cohorts, with operative times consistent with the literature on other robotic platforms. This study demonstrated the practicality and safety of this robotic approach, even when adopted by a surgeon new to robotic techniques. Further studies are needed to evaluate the long-term outcomes and to confirm the results in a larger population.

## Supplementary Information

Below is the link to the electronic supplementary material.

Supplementary material 1 (mp4 10,03,048 KB)

## Data Availability

The datasets generated during and/or analyzed during the current study are available from the corresponding author on reasonable request.

## Abbreviations

BMI: Body mass index
C-D: Clavien-Dindo
LAP: Laparoscopic surgical procedure
IH: Inguinal hernia
IHR: Inguinal hernia repair
IQR: InterQuartile range
POD: Postoperative day
OR: Operating room
RAS: Robot-assisted surgery
rTAPP: Robot-assisted transabdominal preperitoneal inguinal hernia repair
SD: Standard deviation
